# Deficient hippocampal insulin signaling and augmented Tau phosphorylation is related to obesity- and age-induced peripheral insulin resistance: a study in Zucker rats

**DOI:** 10.1186/1471-2202-15-111

**Published:** 2014-09-25

**Authors:** Andrea Špolcová, Barbora Mikulášková, Katarína Kršková, Lucia Gajdošechová, Štefan Zórad, Rafał Olszanecki, Maciej Suski, Beata Bujak-Giżycka, Blanka Železná, Lenka Maletínská

**Affiliations:** Institute of Organic Chemistry and Biochemistry, Prague, 166 10 Czech Republic; Institute of Experimental Endocrinology, Bratislava, 833 06 Slovakia; Jagiellonian University Medical College, Chair of Pharmacology, Krakow, 310 08 Poland

**Keywords:** Zucker fa/fa rats, Insulin resistance, Obesity, GSK-3β, Tau protein

## Abstract

**Background:**

Insulin signaling and Tau protein phosphorylation in the hippocampi of young and old obese Zucker fa/fa rats and their lean controls were assessed to determine whether obesity-induced peripheral insulin resistance and aging are risk factors for central insulin resistance and whether central insulin resistance is related to the pathologic phosphorylation of the Tau protein.

**Results:**

Aging and obesity significantly attenuated the phosphorylation of the insulin cascade kinases Akt (protein kinase B, PKB) and GSK-3β (glycogen synthase kinase 3β) in the hippocampi of the fa/fa rats. Furthermore, the hyperphosphorylation of Tau Ser396 alone and both Tau Ser396 and Thr231 was significantly augmented by aging and obesity, respectively, in the hippocampi of these rats.

**Conclusions:**

Both age-induced and obesity-induced peripheral insulin resistance are associated with central insulin resistance that is linked to hyperTau phosphorylation. Peripheral hyperinsulinemia, rather than hyperglycemia, appears to promote central insulin resistance and the Tau pathology in fa/fa rats.

## Background

Insulin resistance (IR) is a state during which a higher than normal insulin level is required for glucose homeostasis. IR occurs in the periphery and in the brain, where it has recently been linked to the hyperphosphorylation of the neuronal cytoskeleton protein Tau [1], which is symptomatic for Alzheimer’s neurodegeneration. After the glucose homeostasis is disturbed, an increase in the glucose level indicates the onset of type-2 diabetes (T2D).

In several clinical studies, T2D was found to increase the risk of Alzheimer’s disease (AD) [2]. In the postmortem brains of both T2D and sporadic AD patients, central resistance to insulin was documented by attenuated insulin signaling, namely via a decreased phosphorylation of the insulin cascade kinases PDK1 (3-phosphoinositide-dependent protein kinase-1), Akt (protein kinase B, PKB), and GSK-3β (glycogen synthase kinase 3β), and this effect was more pronounced in patients with both T2D and AD [3]. GSK-3β acts as both the insulin cascade kinase and the primary kinase phosphorylating Tau [4, 5]. The phosphorylation of Ser9 in GSK-3β by Akt inhibits the kinase activity of GSK-3β [6, 7], and the attenuated phosphorylation of Ser9 logically increases the kinase activity of GSK-3β toward Tau. Central insulin resistance is linked to a hyperphosphorylation of Tau through GSK-3β [8].

Severe hyperinsulinemia and hyperglycemia, as well as the hyperphosphorylation of Ser199/202, Thr231, and Ser396 in Tau, were found to increase progressively with age in the hippocampi of db/db mice with impaired leptin receptor signaling, a rodent model of T2D [9]. An augmented phosphorylation of Ser396 in the hippocampal Tau of db/db mice was later confirmed by another research team [10]. However, in whole-brain samples of db/db mice with fully developed T2D, changes in the insulin receptors and GSK-3β phosphorylation were not found [11].

Similar to db/db mice, Zucker fatty fa/fa rats have a genetically homozygous leptin receptor mutation that results in leptin dysfunction. Zucker fa/fa rats suffer from obesity induced by hyperphagia, severe hyperlipidemia, and hyperinsulinemia, resulting in IR in the liver, muscle, and adipose tissue [12–14]. The IR in fa/fa rats is established prior to adulthood, at the age of 7 weeks [15]. Unlike db/db mice, fa/fa rats are normoglycemic or have only slightly elevated glucose levels and do not develop diabetes [12, 13].

In this study, insulin signaling and Tau phosphorylation were followed in the hippocampi of 12- (young) and 33-week-old (old) obese Zucker fa/fa rats and their lean controls to verify the hypothesis that peripheral insulin resistance resulting from obesity and/or old age represents a risk factor for central insulin resistance and that such possible central IR is linked to the pathologic phosphorylation of Tau protein. In short, we aimed to determine whether IR with hyperinsulinemia but normoglycemia is associated with a risk of Tau protein pathology in the hippocampus.

## Results

### Metabolic parameters

**Figure 1:**
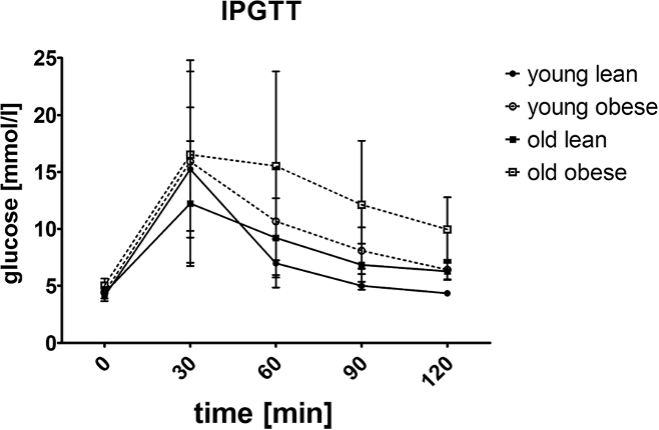
**Graph of inraperitoneal glucose tolerance test.** Before the IPGTT rats were fasted overnight. The intraperitoneal injection of 50% dextrose at dose 2 g/kg was administered and glucose was measured in the tail vein using a glucometer. Data are mean ± SD (n = 6 rats per group). Statistical analysis was calculated by two-way ANOVA, Bonferroni post hoc test.

In old age, both the fa/fa rats and controls developed severe obesity compared with the relevant young controls (F_(1,20)_ = 466.52; p < 0.001). The fa/fa rats also showed a significantly higher body weight than did the age-matched controls (F_(1,20)_ = 236.30; p < 0.001) (Table 1). As expected, obesity in fa/fa rats resulting from impaired leptin receptor signaling was manifested by hyperleptinemia; thus, a significant age and fa/fa genotype interaction exists (F_(1,20)_ = 12.36; p < 0.01), and a subsequent Bonferroni *post-hoc* test revealed an increase in the plasma leptin levels in young fa/fa rats compared with young controls (p < 0.01); this increase was more pronounced in old fa/fa rats compared with old controls (p < 0.001) (Table 1). Obesity was accompanied by hyperinsulinemia. There were significant effects of the fa/fa genotype (F_(1,20)_ = 71.66; p < 0.001) and age (F_(1,20)_ = 13.94; p = 0.001), as well as an age x fa/fa genotype interaction (F_(1,20)_ = 7.99; p = 0.01) with plasma insulin. Significant hyperinsulinemia in fa/fa rats was represented by extreme insulin levels that reached 12-fold (p < 0.001) at 12 weeks of age and 9-fold at 33 weeks of age (p < 0.001) in lean age-matched controls. The glucose levels in all rats were similar and did not exceed normal values (Table 1). Quantitative insulin sensitivity check index (QUICKI) was significantly decreased in both 12-week-old obese (p < 0.05) and 33-week-old obese rats (p < 0.05) compared to age-matched lean controls. Both age (p = 0.002) and fa/fa genotype (p = 0.012) were accompanied with higher and longer lasting rise in glycaemia during glucose tolerance test as revealed by general linear model for repeated measures. However, there was no interaction between these factors (Figure 1). The impairment in glucose tolerance was assessed also using parameter of 2-h glycemia during IPGTT. This impairment was observed with respect to age (F_(1,20)_ = 19.30; p < 0.001) as well as to fa/fa genotype (F_(1,20)_ = 21.44; p < 0.001). There was no detected effect of an age x fa/fa genotype interaction. The area under the curve of the glucose level during IPGTT was found to increase due only to the fa/fa genotype (F_(1,20)_ = 5.41; p < 0.05).

**Table 1:** **Metabolic parameters of fa/fa (obese) rats and their age matched controls**

Rats	Weight [g]	Leptin [ng/ml]	Insulin [ng/ml]	Glucose [mmol/l]	QUICKI
Young control	257 ± 14,17	2,02 ± 1,23	0,50 ± 0,24	6,00 ± 0,42	0,537 ± 0,071
Young fa/fa	386 ± 13,68***	36,72 ± 5,20**	6,26 ± 2,14**	6,27 ± 0,63	0,234 ± 0,008*
Old control	457 ± 21,38***	6,33 ± 1,72	1,43 ± 0,38	6,38 ± 0,43	0,274 ± 0,010
Old fa/fa	683 ± 48,31^###, ooo^	88,66 ± 32,71^###, ooo^	12,96 ± 4,50^###, ooo^	6,80 ± 0,51	0,216 ± 0,007^o^

Data are mean ± SD, n = 6 animals per group. Significance is *P < 0,05, **P < 0,01 and ***P < 0,001 (*vs. young control rats, ^#^vs. young fa/fa rats, ^o^vs. old control rats) using two-way ANOVA, Bonferroni post hoc test.

Significance P<0.05, P<0.01, or P<0.001 is illustrated by one, two, or three symbols, respectively. Particular symbols are for particular groups compared.

Dyslipidemia in young and old fa/fa rats was noticed based on the serum lipid parameters. Both total cholesterol (F_(1,20)_ = 120.38; p < 0.001) and cholesterol/HDL ratio (F_(1,20)_ = 23.55; p < 0.001) were significantly increased in fa/fa rats compared with lean rats (Table 2). Statistical analysis also revealed that age significantly affected the plasma total cholesterol (F_(1,20)_ = 58.30; p < 0.001) and cholesterol/HDL ratio (F_(1,20)_ = 4.38; p < 0.05). A significant age x fa/fa genotype interaction significantly affects the plasma total cholesterol (F_(1,20)_ = 42.83; p < 0.001) and cholesterol/HDL ratio (F_(1,20)_ = 6.26; p < 0.05). As revealed in the post-hoc test, both the plasma total cholesterol were significantly increased in young and old fa/fa rats compared with their age-matched lean controls (p < 0.001). An age-dependent increase in this lipid parameter was observed only in fa/fa rats (p < 0.001). The cholesterol/HDL ratio was increased in old fa/fa rats compared with their lean age-matched rats (p < 0.001) and in old fa/fa rats vs. young fa/fa rats (p < 0.01). In the case of plasma triglycerides levels, a significant elevation was noticed only in 33-week-old obese Zucker rats compared to lean rats of the same age.

**Table 2:** **Levels of lipids in blood serum of fa/fa (obese) rats and their age matched controls**

Rats	Cholesterol [mmol/l]	Triglycerides [mmol/]	Cholesterol/HDL
Young control	2,25 ± 0,23	0,783 ± 0,117	1,605 ± 0,066
Young fa/fa	3,40 ± 0,41**	2,917 ± 0,703	1,988 ± 0,214
Old control	2,53 ± 0,15	0,783 ± 0,098	1,539 ± 0,067
Old fa/fa	7,08 ± 1,17^###, ooo^	4,000 ± 0,802^oo^	2,736 ± 0,763^##, ooo^

Data are mean ± SD, n = 6 animals per group. Significance is *P < 0,05, **P < 0,01 and ***P < 0,001 (*vs. young control rats, ^#^vs. young fa/fa rats, ^o^vs. old control rats) using two-way ANOVA, Bonferroni post hoc test.

Significance P<0.05, P<0.01, or P<0.001 is illustrated by one, two, or three symbols, respectively. Particular symbols are for particular groups compared.

### Insulin signaling cascade in the hippocampus

**Figure 2:**
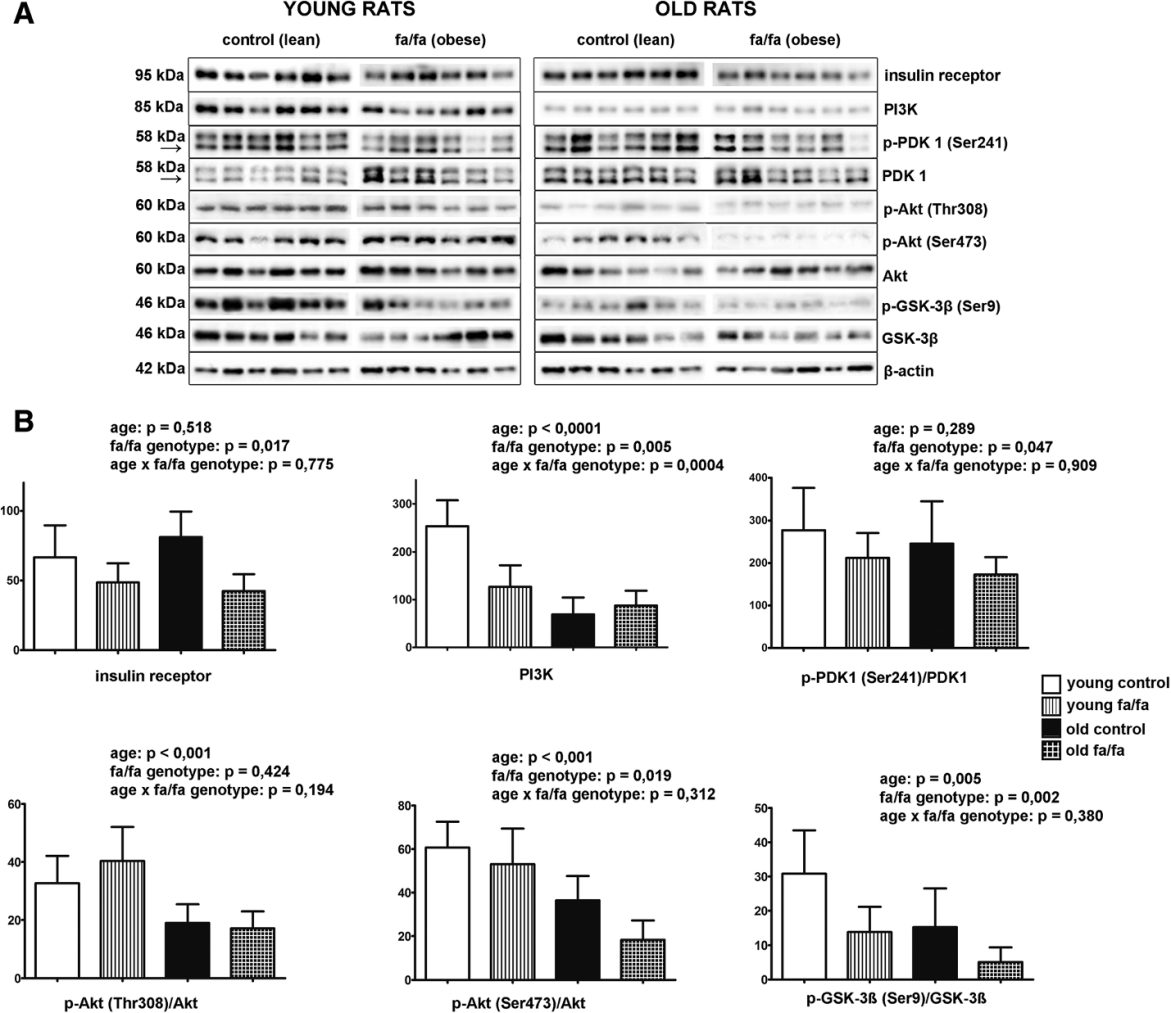
**Insulin signaling cascade in hippocampi of fa/fa rats and their control. A** Western blots of the proteins involved in insulin signaling cascade using specific antibodies (n = 6 rats per group) **B** Densitometric quantification of the western blots normalized to beta-actin: data are mean ± SD (n = 6 rats per group). Statistical analysis was calculated by two-way ANOVA, Bonferroni post hoc test.

Regarding the insulin cascade, a two-way ANOVA revealed a significant main effect of the fa/fa genotype (F_(1,20)_ = 6.82; p < 0.05) on insulin receptor protein expression. In Zucker fatty rats, obesity was associated with lower hippocampal insulin receptor protein levels (Figure 2). However, aging did not affect hippocampal insulin receptor protein expression (F_(1,20)_ = 0.43; p < 0.52). There was no significant interaction between age and genotype (F_(1,20)_ = 0.78; p < 0.08).

Age had a main effect on decreasing the level of PI3 kinase (PI3K) (F_(1,20)_ = 42.03; p < 0.001). The level of PI3K was also attenuated due to the fa/fa phenotype (F_(1,20)_ = 9.84; p < 0.001) (Figure 2). The two-way ANOVA revealed a significant interaction between age and genotype (F_(1,20)_ = 17.77; p < 0.001). Bonferroni’s post-hoc test revealed significantly decreased levels of PI3K in young obese rats compared with young lean rats (p < 0.001) and in old lean rats compared with young lean rats (p < 0.001). No significant differences were observed between old obese and old lean rats.

As determined by the two-way ANOVA, there was significant main effect of fa/fa genotype (F_(1,20)_ = 5.00; p < 0.05) on the phosphorylation of PDK1 Ser241 in the hippocampus (Figure 2). Obesity decreased PDK1 Ser241 phosphorylation in the hippocampi of Zucker fa/fa rats. Neither a significant main effect of age nor an interaction between age and genotype was detected (Figure 2).

The two-way ANOVA revealed a significant main effect of age (F_(1,20)_ = 27.11; p < 0.001) on the phosphorylation of Akt Thr308 (Figure 2). Aging significantly attenuated the hippocampal phosphorylation of Akt Thr308 in both fa/fa and lean rats. Neither a significant effect of genotype (F_(1,20)_ = 0.67; p < 0.42) nor an interaction between age and genotype (F_(1,20)_ = 1.80; p < 0.19) was noted. Regarding the phosphorylation of Akt Ser473, significant main effects of age (F_(1,20)_ = 31.10; p < 0.001) and the fa/fa genotype (F_(1,20)_ = 6.51; p < 0.05) were observed. Both aging and obesity attenuated the phosphorylation of Akt Ser473 (Figure 2). There was no significant interaction between age and the fa/fa genotype (F_(1,20)_ = 1.08; p < 0.31).

Similarly, significant main effects of age (F_(1,20)_ = 9.84; p < 0.01) and the fa/fa genotype (F_(1,20)_ = 12.26; p < 0.01) (Figure 2) on the phosphorylation of GSK-3β Ser9 were observed. Both aging and obesity reduced the phosphorylation of GSK-3β Ser9 (Figure 2). No significant interaction between these factors was detected (F_(1,20)_ = 0.81; p < 0.38).

### Abnormal phosphorylation of tau protein in the hippocampus

**Figure 3:**
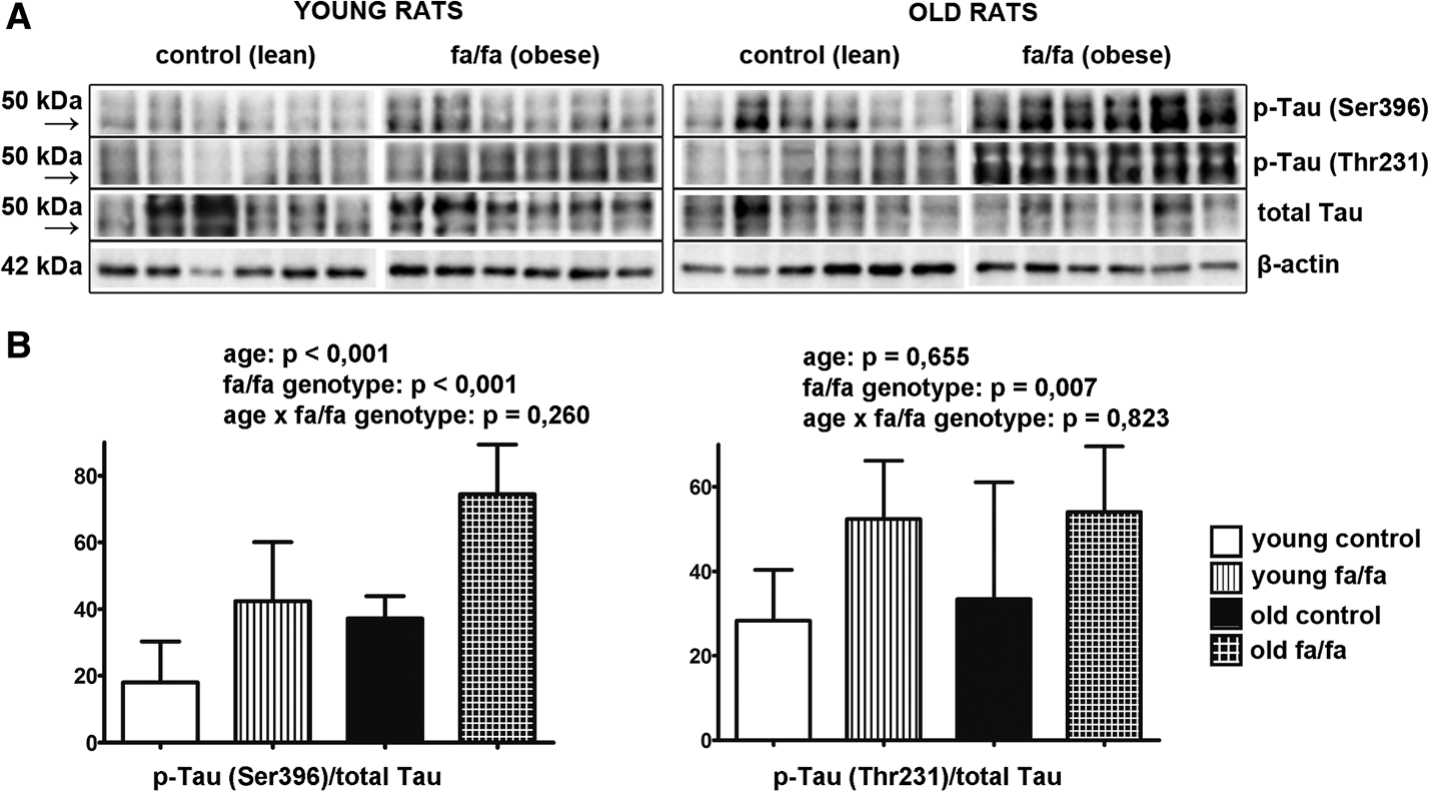
**Hyperphosphorylation of Tau protein on different epitopes in hippocampi of fa/fa rats and their control. A** Western blots of phosphorylation of Tau protein on the epitopes Ser396 and Thr231 (n = 6 rats per group) **B** Densitometric quantification of western blots normalized to β-actin: data are mean ± SD (n = 6 rats per group). Statistical analysis was calculated by two-way ANOVA, Bonferroni post hoc test.

Regarding the phosphorylation of tau at Ser396 in the hippocampus, main effects of age (F_(1,20)_ = 21.55; p < 0.001) and genotype (F_(1,20)_ = 31.16; p < 0.001) were found. Both of these factors increased the phosphorylation of hippocampal tau at Ser396 (Figure 3). There was no significant interaction between age and the fa/fa genotype (F_(1,20)_ = 1.35; p < 0.26). The two-way ANOVA revealed a significant main effect of fa/fa genotype (F_(1,20)_ = 8.86; p < 0.01) on Tau Thr231 phosphorylation (Figure 3). Obesity significantly increased the phosphorylation of hippocampal Tau Thr231. Neither a significant effect of age (F_(1,20)_ = 0.21; p < 0.66) nor an interaction between age and genotype (F_(1,20)_ = 0.05; p < 0.82) was noted.

## Discussion and conclusions

This study revealed attenuated insulin signaling and increased hyperphosphorylation of Tau (at Ser396 and Thr231) in the hippocampi of fa/fa rats exhibiting peripheral insulin resistance or advanced age.

Both the fa/fa rats in this study and the db/db mice in a previous work [9] exhibited impaired leptin signaling, hyperinsulinemia and blood insulin levels approximately 10-fold higher than the normal values. These data indicate that peripheral insulin resistance developed at an early age (8 and 12 weeks) in the db/db mice and the fa/fa rats, respectively. IR in the fa/fa rats was indirectly demonstrated using the QUICKI test. Dyslipidemia in the fa/fa rats could be linked to peripheral IR.

The fa/fa rats were normoglycemic even at an advanced age (33 weeks), whereas 8-week-old db/db mice had glucose levels double those of the control db + mice [9]. Glucose or glucosamine availability is considered to determine the degree of GlcNAcylation of the serines and threonines in the Tau protein that can be phosphorylated [16]. The attenuation of GlcNAcylation in favor of the augmented phosphorylation of the Tau protein has been described as a possible mechanism of Tau pathology [17–19]. On the other hand, healthy mice deprived of food for one to three days (which likely resulted in lower than normal glucose levels) exhibited reversible phosphorylation of hippocampal Tau Ser396 [20]. The fa/fa rats in the present study exhibited an obvious increase in the hyperphosphorylation of hippocampal Tau protein in the normoglycemic state. This finding supports the hypothesis that insulin ineffectiveness, rather than extreme glucose levels, is linked to Tau hyperphosphorylation.

In this study, the obese rats had significantly reduced hippocampal levels of insulin receptor and PI3 kinase protein. Statistically significantly attenuated phosphorylation of both Akt Thr308 and Ser473 was detected in old-age rats, and this effect was more pronounced in fa/fa rats. A similar trend was found for the phosphorylation of Ser9 in GSK3/β. GSK-3β, a kinase common in insulin cascading and Tau phosphorylation, is constitutively active in resting neurons, and its activity is negatively affected by Ser9 phosphorylation [6, 7]. GSK-3β is the primary Tau kinase that hyperphosphorylates Tau [21–23], with Ser199, Thr231, Ser396, and Ser413 as the predominant targets [24]. Cavallini et al. [25] identified GSK-3β and also GSK-3α and MAPK13 as the most active out of 352 kinases overexpressing both Tau kinases and Tau protein [25]. Besides Akt and GSK-3β, extracellular signal-regulated kinase (ERK), is involved both in insulin signaling and Tau phosphorylation. However, there were found no differences in ERK1/2 phosphorylation between the groups in this study (not shown).

The Ser396 and Thr231 phosphorylations investigated in this study are essential in Tau protein pathology for the following reasons. Ser396 is directly phosphorylated by GSK-3β without priming (previous phosphorylation of another Ser or Thr on the Tau protein) [26]. Moreover, a positive relationship between the phosphorylation of Tau Ser396 in the cerebrospinal fluid and the severity of the disease was found in AD patients [27]. Additionally, NIRCO (neuron specific knock-out) mice without insulin receptors in brain neurons showed an increase in the phosphorylation of Tau Thr231, which resulted from the attenuated phosphorylation of Akt Ser473 and GSK-3β Ser9 [5]. Cavallini et al. [25] specified the main phosphorylation sites of Tau as Ser202, Thr231, Ser235, and Ser396/404. In addition to Ser396 and Thr231, we examined Tau phosphorylation at Ser212/214 and Ser 202 in all the groups, but the results were inconsistent.

Inefficient leptin signaling in fa/fa rats could contribute to Tau hyperphosphorylation because leptin has been reported to prevent Tau phosphorylation in neuronal cells via the activation of AMP-dependent kinase [28]. This process was later found to be stress dependent [29], making its role in Tau pathology unclear.

This study demonstrated that the phosphorylation of Ser396 and Thr231 in hippocampal Tau was related to the fa/fa obese phenotype; an interaction with the rats’ age was found for Ser396 only. Analogously, in the hippocampi of db/db mice with non-functioning leptin receptors and severe IR, Tau phosphorylation at Ser 199/202, Thr231, and Ser396 was found to progress with age. Unfortunately, the previous study of db/db mice [9] did not provide data on hippocampal insulin signaling.

In a human study [3], attenuated insulin signaling was inversely correlated with increased Tau hyperphosphorylation in the frontal cortex of T2D patients, and this correlation was more pronounced in T2D patients with AD co-morbidity. However, data on insulin, glucose, and lipid levels were not accessible because of postmortem sampling. Nevertheless, both impaired insulin signaling and Tau hyperphosphorylation in the brain were obvious in both the human study [3] and this rat study.

Peripheral IR in old-age rats appeared to result in central insulin resistance and Tau hyperprotein phosphorylation in the hippocampus. This effect was more pronounced in obese fa/fa rats, which are prone to obesity-induced IR. Based on the normoglycemic state of the IR fa/fa rats, we conclude that a pre-T2D state with IR and normoglycemia is associated with an increased risk of central pathological IR and Tau phosphorylation. The precise mechanism and the role of leptin signaling should be elucidated.

## Methods

### Animals

This investigation was conducted in accordance with ethical standards of the Declaration of Helsinki. This study conformed to national and international guidelines and was approved by the authors’ institutional review board. All experimental procedures and animal care were carried out according to the Jagiellonian University Ethical Committee on Animal Experiments (No 75/2011).

Old (33 weeks old) and young (12 weeks old) male obese Zucker *fa/fa* rats and their age-matched lean controls (n = 6 per each group) were maintained at Jagellonian University in Krakow, Poland. Lean individuals (dominant homozygotes *Fa/Fa* or heterozygotes *Fa/fa*) served as lean controls for the obese *fa/fa* rats. The animals had free access to food and water.

The overnight-fasted rats were euthanized by decapitation. The blood glucose was measured at Synlab (Bratislava, Slovakia) using the multi-analyzer COBAS Integra 800 (Roche Diagnostics Ltd., Rotkreuz, Switzerland), and the serum leptin and insulin levels were determined using RIA kits (Millipore, USA) following the manufacturer’s instructions. The measurements of the serum lipids were performed in the Laboratory Diagnostics Unit of The University Hospital in Krakow using commercially available kits (Roche Molecular Diagnostics, Pleasanton, CA, USA). The quantitative insulin sensitivity check index (QUICKI) [30] was calculated as QUICKI = 1/[(log(I_0_) + log(G_0_)], where I_0_ is the fasting plasma insulin level (microunits per mL), and G_0_ is the fasting blood glucose level (milligrams per dL).

### Intraperitoneal glucose tolerance test

All rats were subjected to an intraperitoneal glucose tolerance test (IPGTT) 2 days prior to euthanasia and after a 16-hour-long overnight fast. The rats were administered an intraperitoneal injection of 50% dextrose at a dose of 2 g/kg body weight. The blood glucose was measured using a glucometer (Accu-Chek Active, Roche Diagnostics, Germany) in the tail vein blood prior to and 30, 60, 90, and 120 min after glucose administration.

### Tissue preparation for western blotting

The dissected hippocampi were homogenized in a glass microhomogenizer using lysis buffer (62.5 mM Tris–HCl buffer with pH 6.8, 1% deoxycholate, 1% Triton X-100, 50 mM NaF, 1 mM Na_3_VO_4_, and Complete Protease Inhibitor (Roche, Switzerland). The lysates were sonicated for 10 min and boiled for 10 minutes. The samples for electrophoresis at 1 μg/μl were diluted with a Laemmli sample buffer containing 50 mM NaF and 1 mM Na_3_VO_4_.

### Antibodies

The following primary antibodies were used: insulin receptor rabbit mAb, PI3 kinase rabbit Ab, phospho-PDK1 (Ser241) rabbit mAb, PDK1 rabbit mAb, phospho-Akt (Thr308) rabbit mAb, phospho-Akt (Ser473) rabbit mAb, Akt rabbit mAb, phospho-GSK-3β (Ser9) rabbit mAb, and GSK-3 β rabbit mAb (all from Cell Signaling Technology, Beverly, MA, USA); phosphoTau (Ser396) rabbit mAb and phosphoTau (Thr231) rabbit mAb (clone PHF13.6 and PHF-6, respectively, both from Invitrogen, NY, USA); CTer mouse mAb for total Tau protein (gift from Dr. M.-C. Galas, Inserm U837, Lille, France); and beta-actin mouse mAb (from Sigma Aldrich). The following secondary antibodies were used: anti-mouse IgG HRP-linked antibody and anti-rabbit IgG HRP-linked antibody (both from Cell Signaling Technology, Beverly, MA, USA).

### Western blotting

Samples of 2–15 μg total protein were subjected to 4/10% SDS-PAGE and transferred onto nitrocellulose (BIO-RAD) or polyvinylidene difluoride (Sigma Aldrich) membranes. The blots were blocked in 5% non-fat milk or 3% BSA in a TBS/Tween buffer (20 mM Tris, 136 mM NaCl, 0.1% Tween-20) with 50 mM NaF and 1 mM Na_3_VO_4_, incubated with the appropriate primary antibody, then incubated with the HRP-linked secondary antibody and developed using the SuperSignal West Femto maximum sensitivity substrate (Pierce, Rockford, IL, USA) following the manufacturer’s instructions. The bands were visualized using the ChemiDoc™ System (BIO-RAD, Hercules, CA, USA) and were quantified using Image Lab Software (BIO-RAD, Hercules, CA, USA). The band intensities were normalized using actin as an internal loading compound, and the ratios of the intensity of the band with the phosphorylated protein and the intensity of the band with the total level of protein were calculated.

### Statistical analysis

The data were analyzed using IBM SPSS 19 Software and are presented as the means ± SD. The data were tested for normality by Shapiro-Wilk test. Normally distributed data were analysed by two-way analysis of variance with interaction with factors of age and fa/fa genotype. Non-normally distributed data were subjected to natural logarithm transformation followed by two-way ANOVA (insulin). Data without normal distribution despite the use of above mentioned transformation were analysed by non-parametric Kruskal-Wallis test (QUICKI, triglycerides). General Linear Model for Repeated Measures was used to evaluate differences in glycaemia during IPGTT. Total area under the curve (AUC) was calculated to describe increment of plasma glucose levels after exogenous glucose load. The overall level of statistical significance was p < 0.05.

## References

[1] Deng Y, Li B, Liu Y, Iqbal K, Grundke-Iqbal I, Gong CX (2009). Dysregulation of insulin signaling, glucose transporters, O-GlcNAcylation, and phosphorylation of tau and neurofilaments in the brain: Implication for Alzheimer’s disease. Am J Pathol.

[2] Kopf D, Frölich L (2009). Risk of incident Alzheimer’s disease in diabetic patients: a systematic review of prospective trials. J Alzheimers Dis.

[3] Liu Y, Liu F, Grundke-Iqbal I, Iqbal K, Gong CX (2011). Deficient brain insulin signalling pathway in Alzheimer’s disease and diabetes. J Pathol.

[4] Hong M, Lee VM (1997). Insulin and insulin-like growth factor-1 regulate tau phosphorylation in cultured human neurons. J Biol Chem.

[5] Schubert M, Gautam D, Surjo D, Ueki K, Baudler S, Schubert D, Kondo T, Alber J, Galldiks N, Küstermann E, Arndt S, Jacobs AH, Krone W, Kahn CR, Brüning JC (2004). Role for neuronal insulin resistance in neurodegenerative diseases. Proc Natl Acad Sci U S A.

[6] Sutherland C, Leighton IA, Cohen P (1993). Inactivation of glycogen synthase kinase-3 beta by phosphorylation: new kinase connections in insulin and growth-factor signalling. Biochem J.

[7] Cross DA, Alessi DR, Cohen P, Andjelkovich M, Hemmings BA (1995). Inhibition of glycogen synthase kinase-3 by insulin mediated by protein kinase B. Nature.

[8] Takashima A (2006). GSK-3 is essential in the pathogenesis of Alzheimer’s disease. J Alzheimers Dis.

[9] Kim B, Backus C, Oh S, Hayes JM, Feldman EL (2009). Increased tau phosphorylation and cleavage in mouse models of type 1 and type 2 diabetes. Endocrinology.

[10] Li J, Deng J, Sheng W, Zuo Z (2012). Metformin attenuates Alzheimer’s disease-like neuropathology in obese, leptin-resistant mice. Pharmacol Biochem Behav.

[11] Jolivalt CG, Lee CA, Beiswenger KK, Smith JL, Orlov M, Torrance MA, Masliah E (2008). Defective insulin signaling pathway and increased glycogen synthase kinase-3 activity in the brain of diabetic mice: parallels with Alzheimer’s disease and correction by insulin. J Neurosci Res.

[12] http://www.criver.com

[13] http://www.harlan.com

[14] Shiota M, Printz RL (2012). Diabetes in Zucker diabetic fatty rat. Methods Mol Biol.

[15] Di Nardo F, Burattini R, Cogo CE, Faelli E, Ruggeri P (2009). Age-related analysis of insulin resistance, body weight and arterial pressure in the Zucker fatty rat. Exp Physiol.

[16] Walgren JL, Vincent TS, Schey KL, Buse MG (2003). High glucose and insulin promote O-GlcNAc modification of proteins, including alpha-tubulin. Am J Physiol Endocrinol Metab.

[17] Dias WB, Hart GW (2007). O-GlcNAc modification in diabetes and Alzheimer’s disease. Mol Biosyst.

[18] Deng Y, Li B, Liu F, Iqbal K, Grundke-Iqbal I, Brandt R, Gong CX (2008). Regulation between O-GlcNAcylation and phosphorylation of neurofilament-M and their dysregulation in Alzheimer disease. FASEB J.

[19] Lefebvre T, Dehennaut V, Guinez C, Olivier S, Drougat L, Mir AM, Mortuaire M, Vercoutter-Edouart AS, Michalski JC (2010). Dysregulation of the nutrient/stress sensor O-GlcNAcylation is involved in the etiology of cardiovascular disorders, type-2 diabetes and Alzheimer’s disease. Biochim Biophys Acta.

[20] Yanagisawa M, Planel E, Ishiguro K, Fujita SC (1999). Starvation induces tau hyperphosphorylation in mouse brain: implications for Alzheimer’s disease. FEBS Lett.

[21] Ishiguro K, Omori A, Takamatsu M, Sato K, Arioka M, Uchida T, Imahori K (1992). Phosphorylation sites on tau by tau protein kinase I, a bovine derived kinase generating an epitope of paired helical filaments. Neurosci Lett.

[22] Yamaguchi H, Ishiguro K, Uchida T, Takashima A, Lemere CA, Imahori K (1996). Preferential labeling of Alzheimer neurofibrillary tangles with antisera for tau protein kinase (TPK) I/glycogen synthase kinase-3 beta and cyclin-dependent kinase 5, a component of TPK II. Acta Neuropathologica.

[23] Hooper C, Killick R, Lovestone S (2008). The GSK3 hypothesis of Alzheimer’s disease. J Neurochem.

[24] Michel G, Mercken M, Murayama M, Noguchi K, Ishiguro K, Imahori K, Takashima A (1998). Characterization of tau phosphorylation in glycogen synthase kinase-3beta and cyclin dependent kinase-5 activator (p23) transfected cells. Biochim Biophys Acta.

[25] Cavallini A, Brewerton S, Bell A, Sargent S, Glover S, Hardy C, Moore R, Calley J, Ramachandran D, Poidinger M, Karran E, Davies P, Hutton M, Szekeres P, Bose S (2013). An unbiased approach to identifying tau kinases that phosphorylate tau at sites associated with Alzheimer disease. J Biol Chem.

[26] Leroy A, Landrieu I, Huvent I, Legrand D, Codeville B, Wieruszeski JM, Lippens G (2010). Spectroscopic studies of GSK3{beta} phosphorylation of the neuronal tau protein and its interaction with the N-terminal domain of apolipoprotein E. J Biol Chem.

[27] Hu YY, He SS, Wang X, Duan QH, Grundke-Iqbal I, Iqbal K, Wang J (2002). Levels of nonphosphorylated and phosphorylated tau in cerebrospinal fluid of Alzheimer’s disease patients: an ultrasensitive bienzyme-substrate-recycle enzyme-linked immunosorbent assay. Am J Pathol.

[28] Greco SJ, Sarkar S, Johnston JM, Zhu X, Su B, Casadesus G, Ashford JW, Smith MA, Tezapsidis N (2008). Leptin reduces Alzheimer’s disease-related tau phosphorylation in neuronal cells. Biochem Biophys Res Commun.

[29] Salminen A, Kaarniranta K, Haapasalo A, Soininen H, Hiltunen M (2011). AMP-activated protein kinase: a potential player in Alzheimer’s disease. J Neurochem.

[30] Katz A, Nambi SS, Mather K, Baron AD, Follmann DA, Sullivan G, Quon MJ (2000). Quantitative insulin sensitivity check index: a simple, accurate method for assessing insulin sensitivity in humans. J Clin Endocrinol Metab.

